# Control first, tolerance second: biologics as enablers of allergen immunotherapy in severe allergic asthma

**DOI:** 10.3389/falgy.2026.1942188

**Published:** 2026-09-09

**Authors:** B. Jentzsch, A. Hoheisel, K. Gashynova, S. Fähndrich, B. C. Frye, J. A. Alvarado Castillo, S. Müller, I. Cherrez-Ojeda, D. Stolz

**Affiliations:** 1 Clinic of Pneumology, Medical Center-University of Freiburg, Faculty of Medicine, University of Freiburg, Freiburg, Germany; 2 Department of Dermatology, Allergy Research Group, Medical Center, University of Freiburg, Freiburg, Germany; 3 Universidad Espiritu Santo, Samborondon, Ecuador; 4 Respiralab Research Group, Guayaquil, Ecuador; 5 Institute of Allergology, Charité-Universitätsmedizin Berlin, Corporate Member of Freie Universität Berlin and Humboldt-Universität zu Berlin, Berlin, Germany; 6 Fraunhofer Institute for Translational Medicine and Pharmacology ITMP, Immunology and Allergology, Berlin, Germany

**Keywords:** allergen immunotherapy, allergic asthma, biologics, dupilumab, omalizumab, severe asthma, tezepelumab, type 2 inflammation

## Abstract

Despite major advances in the treatment of severe asthma with biologics targeting IgE, IL-5/IL-5R*α*, IL-4R*α*, and thymic stromal lymphopoietin (TSLP), these agents suppress type 2 inflammation without reliably inducing durable allergen-specific tolerance. Allergen immunotherapy (AIT) remains the only disease-modifying option for IgE-mediated respiratory allergy, yet its use in severe asthma has historically been restricted by safety concerns in uncontrolled disease. We propose a “control first, tolerance second” framework, whereby biologic-induced disease control creates a window enabling safe initiation of subcutaneous or sublingual AIT. This narrative review synthesizes the immunological rationale and clinical evidence supporting sequential biologic-AIT combination, with emphasis on omalizumab as the best-studied adjunct and emerging data on dupilumab and tezepelumab. We address biomarker-guided patient selection, treatment sequencing, comorbid allergic rhinitis and chronic rhinosinusitis, corticosteroid-sparing effects, infection risk, airway antiviral immunity, and unresolved questions around long-term remission. The novelty of this framework lies in reframing biologics not merely as symptom-control agents but as facilitators that unlock a previously inaccessible disease-modifying intervention, potentially shifting severe allergic asthma management from control toward durable immunological remission. We conclude that biologic-enabled AIT is a promising but insufficiently defined strategy, and outline the prospective trials, standardized phenotyping, and safety registries needed to establish it as an evidence-based clinical pathway.

## 1 Introduction

Severe asthma is no longer viewed as a single therapeutic entity but as a heterogeneous syndrome requiring systematic confirmation of diagnosis, assessment of modifiable factors, characterization of inflammatory phenotype, and targeted add-on treatment. In patients who remain uncontrolled despite optimized high-dose inhaled corticosteroid-containing therapy and additional controllers, severe asthma management increasingly relies on biologic therapies directed against dominant type 2 inflammatory pathways. Contemporary severe asthma guidance recommends consideration of anti-IgE, anti-IL-5/IL-5R*α*, anti-IL-4R*α*, or anti-TSLP treatment in eligible patients after diagnosis, adherence, inhaler technique, comorbidities, and baseline therapy have been optimized (1–3).

Among severe asthma phenotypes, severe allergic asthma occupies a particularly important position. It is characterized by IgE-mediated sensitization to clinically relevant aeroallergens, variable degrees of eosinophilic inflammation, allergen-triggered symptoms or exacerbations, and frequent coexistence of allergic rhinitis, chronic rhinosinusitis, nasal polyps, or atopic dermatitis. This phenotype is clinically relevant because it sits at the intersection of two major immunomodulatory strategies: biologic pathway suppression and allergen-specific immune tolerance induction.

Biologics have transformed the care of severe asthma by reducing exacerbations, improving symptom control and lung function in selected populations, and reducing the need for maintenance systemic corticosteroids. Yet biologics, despite their profound clinical impact, generally suppress or interrupt inflammatory pathways rather than directly inducing durable allergen-specific tolerance after treatment discontinuation (4).

Allergen immunotherapy, by contrast, is designed to modify the underlying allergic immune response. It is associated with regulatory T-cell activity, IL-10 and TGF-*β* signaling, modulation of allergen-specific IgE, increased blocking antibodies such as IgG4, which results in reduced recruitment or activation of effector cells including mast cells, basophils, and eosinophils. It is therefore widely regarded as the only established disease-modifying intervention for IgE-mediated allergic disease (5–8).

The clinical problem is that allergen immunotherapy is not equally suitable for all patients with allergic asthma. Severe or poorly controlled asthma is a recognized safety boundary, particularly for subcutaneous immunotherapy. Current guidance supports careful use of allergen immunotherapy only when asthma is stable and adequately controlled, especially in severe asthma populations (7, 9, 10).

This creates a paradox: the patients who may have the greatest need for long-term disease modification may initially be poor candidates for this intervention.

This review focuses on this dilemma. We propose that biologics and AIT should not be framed simply as competing options in severe allergic asthma. They may represent complementary and potentially sequential strategies: biologic therapy may create sufficient the clinical stability for selected patients to be reassessed for AIT, while AIT aims to induce allergen-specific immune modification. Whether this sequence improves durable tolerance in biologic-eligible severe asthma remains unproven. This hypothesis-generating can be summarized as: control first, tolerance second.

## 2 Conceptual framework: control first, tolerance second

The proposed framework rests on a distinction between inflammatory control and immune tolerance. Inflammatory control refers to suppression of disease activity: fewer exacerbations, less reliever use, improved lung function, reduced oral corticosteroid exposure, and improved patient-reported outcomes. Immune tolerance refers to a more durable recalibration of allergen-specific immune reactivity, with reduced hyperreactivity to otherwise harmless allergen exposure and persistence of benefit beyond the active treatment period.

In severe allergic asthma, the first therapeutic priority is safety. A patient with recent severe exacerbations, unstable lung function, frequent rescue medication use, or uncontrolled symptoms is not an appropriate candidate for AIT. In this phase, biologic therapy may be used to reduce type 2 inflammatory activity and stabilize disease. Only after stable asthma control has been achieved should allergen immunotherapy be reconsidered in selected individuals with a clearly documented allergen-driven phenotype.

This sequencing strategy is aligned with the logic of contemporary severe asthma care: optimize standard therapy, define phenotype and endotype, use biologics in eligible patients with persistent severe disease, and consider allergen immunotherapy only when asthma is clearly antigen-driven and stable enough for safe allergen exposure under specialist supervision (3, 7, 9–12).

The central question is therefore not simply whether biologics or allergen immunotherapy should be chosen. The more precise question is: can biologic therapy convert selected patients with severe allergic asthma from unsafe or unsuitable candidates for allergen immunotherapy into stable candidates for carefully monitored allergen immunotherapy?

A critical uncertainty must be stated explicitly: it is currently unknown whether biologic therapy enhances, is neutral toward, or impairs the induction of long-term allergen-specific immune tolerance by AIT. The available evidence supports that certain biologics improve AIT safety and tolerability — it does not establish that they augment the tolerogenic immune response. These are distinct questions, and the proposed framework cannot answer the latter on the basis of current evidence.

## 3 Immunological rationale

**Figure 1:**
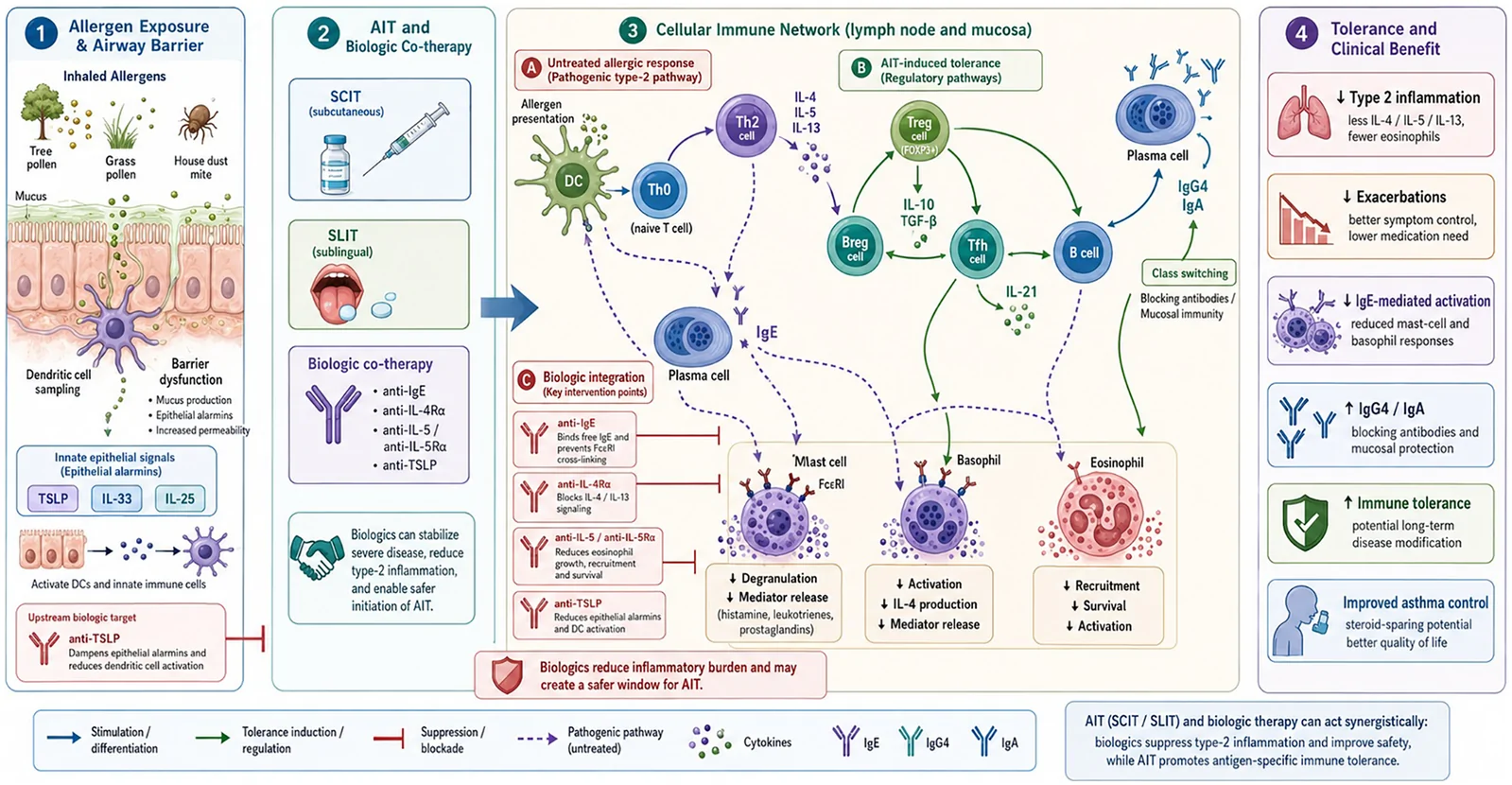
Cellular mechanisms of AIT in severe allergic asthma. 1. Origin of allergic reaction; 2. Treatment options using biologics and AIT; 3. Cellular mechanisms and targets for therapies; 4. Desired treatment outcome. Figure created by the authors using fully licensed versions of BioRender and FigureLabs.

Severe allergic asthma is driven by persistent type 2 immune dysregulation, in which allergen-specific IgE, mast cells, basophils, type 2 innate lymphoid cells, and eosinophils interact to produce airway inflammation, mucus hypersecretion, bronchospasm, and airway remodelling. Central to this process is the allergen-specific Th2-dominated immune response, characterized by IL-4, IL-5, and IL-13 production, IgE class switching, and sustained downstream effector activation (Figure 1). Understanding the immunological consequences of biologic intervention and the immunological prerequisites for successful allergen immunotherapy is essential to the proposed combination strategy (13, 14).

Allergen immunotherapy (AIT) induces immune tolerance through distinct but overlapping mechanisms. Subcutaneous immunotherapy (SCIT) and sublingual immunotherapy (SLIT) both engage dendritic cells in peripheral and mucosal tissues to shift allergen-specific immune responses from Th2 effector dominance toward a regulatory profile, driven primarily by IL-10- and TGF-*β*-producing Tregs and Tr1 cells. Key immunological changes associated with successful AIT include expansion of allergen-specific Foxp3+ Tregs and Tr1 cells, increased IL-10 and TGF-*β* secretion, a shift in antibody production away from IgE and toward IgG4 (and IgG1) antibodies that compete with IgE for allergen binding and thereby limit Fc*ε*RI-mediated effector cell activation and IgE-facilitated antigen presentation, decreased allergen-specific IgE relative to IgG4, mast cell desensitization, and reduced basophil reactivity (Figure 1). Whether these changes reflect durable reprogramming of allergen-specific immune memory or an actively maintained regulatory state remains incompletely understood; however, the persistence of clinical benefit for years after treatment discontinuation—a feature not shared by biologic monotherapy—suggests that AIT engages mechanisms beyond the reversible receptor/antibody-level effects of biologics (5–8, 15).

Omalizumab, a recombinant humanized anti-IgE monoclonal antibody, binds free IgE and downregulates high-affinity IgE receptors (Fc*ε*RI) on mast cells, basophils, and dendritic cells (Figure 1). By reducing available free IgE, omalizumab diminishes allergen-triggered early-phase and late-phase responses, reduces IgE-dependent effector-cell activation, and attenuates IgE-dependent antigen presentation. These effects are directly relevant to allergen immunotherapy safety: the reduction of both, Fc*ε*RI density and free IgE availability lowers the risk of systemic reactions during allergen exposure, providing a theoretical and practical rationale for using omalizumab to enable safer allergen immunotherapy escalation (16–18).

Dupilumab blocks the shared IL-4R*α* subunit of the IL-4 and IL-13 receptors, thereby interrupting both cytokine signalling pathways simultaneously (Figure 1). This suppresses IgE synthesis, reduces IL-13-driven mucus production and airway remodelling, and attenuates eosinophilic airway inflammation. Importantly, dupilumab does not deplete IgE molecules already bound to effector cells, and its effects on allergen-specific IgE kinetics are more gradual than those of omalizumab. Whether the IL-4R*α*-blocking effect impairs or facilitates the IgG4 class-switching that is required for AIT-mediated tolerance induction remains an open immunological question, since IL-4 signalling is involved in both IgE and IgG4 production. IL-4R*α* blockade may either impair AIT-induced tolerance by reducing IgG4 production or compensate through reduced type 2 inflammation and enhanced Treg/IL-10 activity. Determining which mechanism predominates is the key mechanistic priority for dupilumab–AIT combination therapy. This mechanistic ambiguity is one reason why dupilumab-plus-AIT combinations require dedicated clinical study. At present, any statement about the tolerogenic efficacy of AIT under dupilumab background therapy must be considered speculative and remains to be tested in prospective trials (19–23).

Tezepelumab targets thymic stromal lymphopoietin (TSLP), an epithelial alarmin released in response to allergen exposure, respiratory viruses, air pollutants, and other environmental triggers. TSLP acts at the apex of the type 2 inflammatory cascade by activating dendritic cells, promoting Th2 differentiation, and activating innate lymphoid cells. Blocking TSLP with tezepelumab therefore interrupts an upstream step of allergic sensitization and re-stimulation (Figure 1). Whether TSLP blockade also modulates the regulatory immune networks engaged by AIT—particularly Treg induction and IgG4 class switching—is currently unknown. The broad anti-inflammatory effect of TSLP blockade may theoretically reduce the allergen-driven re-stimulation that otherwise maintains persistent type 2 sensitization, potentially lowering the immunological barrier to tolerance induction; however, this hypothesis has not been tested in any clinical study and cannot be assumed to translate into a meaningful tolerogenic benefit in practice (24–26).

Therefore, Tezepelumab may paradoxically both facilitate tolerance and impair AIT-induced regulatory responses by reducing allergen-driven dendritic cell activation. Whether these effects are complementary or inhibitory remains unknown and warrants dedicated experimental investigation.

Anti-IL-5 agents (mepolizumab, depemokimab, reslizumab) and the anti-IL-5R*α* antibody benralizumab reduce circulating and tissue eosinophils with high specificity, but their role in allergen-specific sensitization is more limited. In predominantly eosinophilic asthma with a weaker allergic component, the rationale for combining anti-IL-5 therapy with AIT may be less compelling than in IgE-dominant disease (Figure 1). Nevertheless, eosinophil depletion reduces one major effector arm of the late-phase allergic response and could hypothetically improve the safety profile of allergen immunotherapy escalation in selected patients; this remains unproven, as no published clinical data address the combination of anti-IL-5 agents with AIT in severe asthma (21, 27, 28).

From an immunological perspective, the combination of biologic therapy and AIT is therefore not redundant but potentially complementary: biologics suppress the acute and chronic inflammatory activity that makes AIT unsafe, while AIT engages distinct regulatory mechanisms aimed at durable allergen-specific tolerance (Figure 1). The rationale and clinical evidence are strongest for omalizumab. Direct combination studies also exist for dupilumab and tezepelumab in allergic rhinitis or asthma with rhinitis, but no randomized trial establishes efficacy, safety, or durable tolerance for either agent in biologic-eligible severe asthma. Evidence for anti-IL-5/IL-5R*α* combinations remains mechanistic or background evidence only. All non-omalizumab strategies should therefore be regarded as hypothesis-generating and require prospective validation (8, 16, 19, 24, 29–31).

## 4 Evidence for combining biologics and allergen immunotherapy

### 4.1 Omalizumab and allergen immunotherapy

The combination of omalizumab with allergen immunotherapy is the best-studied biologic–AIT strategy and constitutes the primary clinical evidence base for the proposed framework. The rationale for this combination was recognized early after omalizumab became available, driven by observations that omalizumab reduced systemic reaction risk during SCIT up-dosing and enabled safer escalation in patients exposed to allergen immunotherapy (17, 18, 32, 33).

Several randomized controlled and controlled clinical studies have shown that omalizumab pretreatment can improve the tolerability of accelerated or seasonal immunotherapy protocols in selected patients with allergic rhinitis and comorbid asthma. In a pivotal randomized controlled trial by Massanari et al. (18), co-administration of omalizumab with specific immunotherapy in allergic asthma significantly reduced systemic reactions during immunotherapy build-up compared with immunotherapy alone. Kamin et al. reported similar safety improvements in children receiving omalizumab during specific immunotherapy for seasonal allergic rhinitis. The combination appeared not only to improve safety but also to produce additive reductions in symptom scores, resulted in lower exacerbation rates and rescue medication use compared with either approach alone (17, 18, 32–34). This safety and efficacy signal has since been formally synthesized in a random-effects meta-analysis of 11 randomized controlled trials (901 patients), which found that omalizumab co-administration significantly increased both target maintenance dose achievement (OR 2.43, 95% CI 1.33–4.44) and sustained unresponsiveness to allergen challenge (OR 6.77, 95% CI 2.10–21.80), while significantly reducing severe systemic adverse events (OR 0.32, 95% CI 0.18–0.59), relative to AIT alone (35). Within this pooled analysis, however, the asthma-specific subgroup was limited to two trials (OR 2.09, 95% CI 1.23–3.53), and the sustained-unresponsiveness endpoint was derived exclusively from oral food-immunotherapy trials rather than inhalant AIT for asthma — a distinction addressed further below.

In the context of severe asthma specifically, omalizumab may convert patients who would otherwise be excluded from SCIT consideration into candidates for carefully monitored allergen immunotherapy. Patients treated with omalizumab who achieve sustained asthma control with FEV1 values stably above 70% predicted and minimal exacerbation burden may fulfil the clinical stability criteria required for AIT initiation under specialist supervision. Two more recent randomized controlled trials conducted specifically in adult patients with house dust mite-driven asthma provide direct, if still modest, support for this approach: a three-arm trial (*n* = 52) demonstrated that SCIT plus omalizumab produced greater improvements in ACQ score, exacerbation rate, and inhaled corticosteroid reduction than SCIT alone (36), and a subsequent four-arm, 24-month trial (*n* = 82) confirmed a progressive, significantly greater reduction in inhaled corticosteroid dose and exacerbation frequency with the combination compared with either monotherapy or placebo (37). Neither trial, however, enrolled a population meeting GINA criteria for severe asthma specifically; both were conducted in mild-to-moderate house dust mite–driven asthma, so direct evidence in the severe, biologic-eligible population targeted by this review remains absent. The clinical benefit of subsequently adding AIT in this setting—whether in terms of further allergen-specific symptom reduction, steroid sparing, or long-term remission after omalizumab discontinuation—requires prospective evaluation in adequately powered controlled trials (38–41). It should be noted that the strongest quantitative evidence for durable, treatment-free tolerance (sustained unresponsiveness) with omalizumab-enabled AIT derives from oral food immunotherapy trials, not from inhalant AIT in asthma; extrapolation of a ‘tolerance' endpoint to severe allergic asthma remains, at present, a mechanistic inference rather than an established finding (35).

The evidence base for omalizumab-facilitated AIT therefore represents the only combination strategy with replicated randomized controlled trial data; all other biologic–AIT combinations discussed in this review are supported by mechanistic reasoning, case series, or expert opinion only, and must be clearly distinguished from omalizumab-enabled strategies when communicating the strength of evidence to clinicians or patients (Table 1).

**Table 1:** Key studies informing the biologic–allergen immunotherapy (AIT) combination framework in severe allergic asthma.

First Author, Year [Ref.]	Study Design	Population	n	Key Finding/Outcome	Evidence Level^a^
SECTION 1: Omalizumab + Allergen Immunotherapy					
Casale et al., 2,006 (17)	RCT, DB, PC	Seasonal AR ± asthma	159	Omalizumab pretreatment significantly reduced anaphylaxis during rush SCIT build-up (0 vs. 6.7%)	2 – RCT
Massanari et al., 2010 (18)	RCT, DB, PC	Moderate–severe allergic asthma	248	Significantly fewer systemic reactions with omalizumab + SCIT vs. SCIT alone (2.5 vs. 10.2%/injection); improved ACQ and rescue medication use	2 – RCT
Kopp et al., 2009 (32)	RCT (DUAL study)	Seasonal AR + comorbid asthma (paediatric)	221	Combination superior to SCIT alone: better symptom scores, rescue use, and asthma control	2 – RCT
Kamin et al., 2010 (33)	CCT	Children with AR undergoing SCIT	38	No increase in systemic reactions; safe combination profile in paediatric population	3 – CCT/cohort
Bozek et al., 2023 (36)	RCT, 3-arm	Adults, HDM-driven asthma (mild–mod)	52	SCIT + omalizumab superior to SCIT alone: greater ACQ improvement, lower exacerbation rate, greater ICS reduction at 12 months	2 – RCT
Bozek et al., 2024 (37)	RCT, 4-arm, 24-month	Adults, HDM-driven asthma (mild–mod)	82	Progressive ICS dose reduction and fewer exacerbations with combination vs. monotherapy or placebo over 24 months	2 – RCT
Bozek et al., 2026 (34)	RCT, 3-year, 3-arm	Adults with allergic asthma	–	Sustained ICS reduction and IgE/IgG4 biomarker improvements with combination vs. monotherapy over 3 years	2 – RCT
Zhang et al., 2024 (35)	Meta-analysis (11 RCTs)	Mixed (AR, food allergy, asthma subgroup)	901	Omalizumab + AIT: ↑ maintenance dose achievement (OR 2.43; 95% CI 1.33–4.44), ↑ sustained unresponsiveness (OR 6.77), ↓ severe AEs (OR 0.32). Note: unresponsiveness derived from food OIT trials, not inhalant AIT	1 – Meta-analysis/SR
Lambert et al., 2014 (38)	Retrospective series	Children/young adults with severe asthma excluded from AIT	22	Omalizumab pretreatment enabled AIT initiation without serious systemic reactions in previously excluded patients	4 – Case series/report
SECTION 2: Dupilumab + Allergen Immunotherapy					
Corren et al., 2021 (29)	RCT	Adults with moderate–severe AR	54	Dupilumab + short-course SCIT well tolerated; no increase in systemic reactions; dupilumab improved AR outcomes independently of SCIT	2 – RCT
Hoshino et al., 2022 (30)	Prospective open-label	Adults with asthma + rhinitis on dupilumab	31	HDM-SLIT add-on to dupilumab tolerated; improvements in asthma and rhinitis scores; allergen-specific IgG4 increase observed despite IL-4R*α* blockade	3 – CCT/cohort
Kim et al., 2024 (22)	Retrospective series	Adults with severe atopic dermatitis	24	Combination feasible; IgG4 increases detected despite IL-4Rα blockade; no serious systemic reactions. Not asthma-specific	4 – Case series/report
Tussupbekova et al., 2025 (23)	Case report	Single patient: severe AD + asthma	1	Dupilumab + SCIT tolerated; symptom improvement in AD and asthma. Mechanistic endpoints not reported	4 – Case series/report
SECTION 3: Tezepelumab + Allergen Immunotherapy					
Corren et al., 2023 (31)	RCT (nasal challenge model)	Adults with AR and perennial sensitization	20	Tezepelumab + AIT significantly reduced peak total nasal symptom score vs. AIT alone after allergen challenge; first direct tezepelumab–AIT combination RCT data	2 – RCT
Menzies-Gow et al., 2021 (NAVIGATOR) (24)	Phase 3 RCT	Severe uncontrolled asthma (broad strata)	1,061	Tezepelumab reduced exacerbation rate by 56%. No AIT arm — basis for mechanistic extrapolation only; no data on AIT-specific immune tolerance	2 – RCT

a Evidence levels (Oxford CEBM 2011): 1 = Systematic review/meta-analysis of RCTs | 2 = RCT | 3 = Controlled clinical trial or cohort | 4 = Case series/report | 5 = Expert review/narrative.

AR, allergic rhinitis; AIT, allergen immunotherapy; CCT, controlled clinical trial; DB, double-blind; HDM, house dust mite; ICS, inhaled corticosteroids; OIT, oral immunotherapy; OR, odds ratio; PC, placebo-controlled; RCT, randomized controlled trial; SCIT, subcutaneous immunotherapy; SLIT, sublingual immunotherapy; SR, systematic review.

For dupilumab, only one small RCT (Corren 2021, *n* = 54) and one open-label study (Hoshino 2022) with AIT exist, both outside the severe asthma context. For tezepelumab, one RCT (Corren 2023) used a nasal challenge model, not a clinical AIT protocol in asthma. No clinical combination data exist for anti-IL-5 agents. Evidence in Sections 2–5 should not be interpreted as clinical guidance for routine use.

### 4.2 Dupilumab and allergen immunotherapy

Clinical evidence for dupilumab co-administered with allergen immunotherapy is considerably more limited than for omalizumab, consisting at present exclusively of case series and single case reports in atopic dermatitis — not in severe asthma — and must be regarded as preliminary and hypothesis-generating rather than practice-informing. Dupilumab is approved for severe asthma, atopic dermatitis, chronic rhinosinusitis with nasal polyps, eosinophilic oesophagitis, and prurigo nodularis—conditions frequently co-occurring in patients with severe allergic asthma—and its profound multi-organ anti-inflammatory efficacy has led to its increasingly frequent use in complex atopic patients. Given that some of these patients also receive or may benefit from allergen immunotherapy, real-world co-administration is already occurring, and early observational and case-report data have begun to emerge (19, 20, 22, 23).

A key mechanistic concern with the dupilumab–AIT combination relates to the role of IL-4 in IgG4 class switching. AIT-associated tolerance is partly mediated by a shift from IgE toward IgG4 blocking antibodies, a process in which IL-4 signalling participates. Since dupilumab blocks the IL-4R*α* receptor, it could theoretically attenuate aspects of the class-switching response that contribute to AIT-induced tolerance. Preliminary clinical reports in atopic dermatitis suggest that combined dupilumab and allergen immunotherapy can be feasible and may still be associated with allergen-specific IgG4 dynamics, but the evidence remains small and not asthma-specific. The net immunological balance — between improved disease stability on one hand, and potentially impaired tolerogenic IgG4 class-switching on the other — cannot currently be resolved. Whether dupilumab facilitates, is neutral toward, or actively undermines AIT-induced tolerance remains an open question that requires adequately powered prospective trials before this combination can be recommended in any clinical context. Clinicians considering dupilumab plus AIT outside a trial setting should be explicit with patients that this represents an individualized decision in the absence of supporting evidence (21–23).

### 4.3 Tezepelumab and allergen immunotherapy

No published clinical data exist on the combination of tezepelumab and allergen immunotherapy. The following discussion is therefore entirely mechanistic and speculative, and should not be interpreted as evidence for clinical practice. Tezepelumab was approved for severe asthma on the basis of its upstream mechanism at the epithelial interface and clinical efficacy across broad biomarker strata. The NAVIGATOR trial demonstrated broad efficacy including in patients without elevated blood eosinophils or FeNO, which suggests a wide therapeutic window. Whether this broad suppression of upstream alarmin signalling interferes with, facilitates, or remains neutral toward the immunological mechanisms of AIT is not currently known. The observation that TSLP blockade reduces dendritic cell activation and antigen presentation suggests that it may theoretically modulate the first steps of allergen sensitization and re-sensitization, potentially complementing AIT by reducing the re-stimulatory allergen signal. However, this also raises the question of whether AIT-specific immune programming can be adequately initiated in the context of profound upstream TSLP suppression. These mechanistic questions require dedicated pre-clinical and early-phase clinical investigation before any guidance can be provided. At present, tezepelumab combined with AIT should be considered a research hypothesis only, with no clinical evidence base to support its use outside of a prospective trial setting (24–26).

## 5 Proposed clinical algorithm

**Figure 2:**
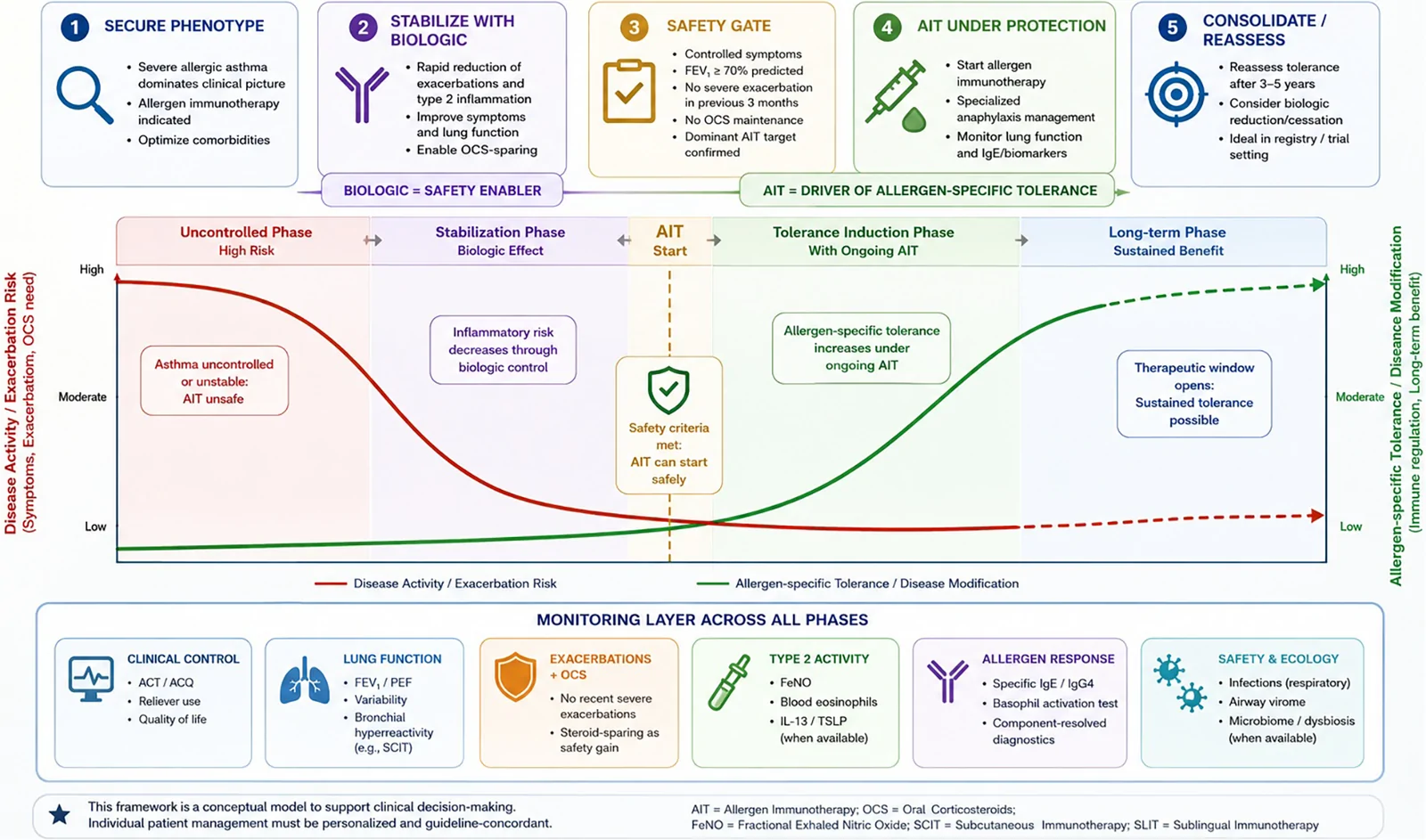
Clinical timeframe of biologics as enablers of allergen immunotherapy in severe allergic asthma: after securing the asthma-phenotype (1) biologics reduce inflammatory risk first (2/3); AIT follows when tolerance induction is feasible (4) and leads to longterm benefit (5). Throughout the timeframe close monitoring is crucial. Figure created by the authors using fully licensed versions of BioRender and FigureLabs.

Based on available evidence and the proposed conceptual framework, a stepwise clinical algorithm can be outlined for the evaluation and management of patients with severe allergic asthma who may be considered for combined biologic and AIT treatment (Figure 2). This algorithm is intended as a clinical framework for expert centers and should not be applied outside specialist allergy and respiratory medicine settings. It is not a substitute for individualized clinical judgement and requires prospective validation (1, 3, 7, 9).

### Step 1: confirm diagnosis and exclude mimics

Objective confirmation of asthma diagnosis with documented bronchodilator reversibility, positive bronchial provocation test, or characteristic peak flow variability. Exclusion of relevant mimics including vocal cord dysfunction, ABPA, hypersensitivity pneumonitis, EGPA, and cardiac causes of dyspnoea. Assessment and optimization of modifiable factors: adherence to inhaled therapy, inhaler technique, smoking cessation, obesity management, occupational exposures, and gastro-oesophageal reflux (1–3).

### Step 2: characterize allergic phenotype

Skin prick testing and/or allergen-specific IgE measurement to identify clinically relevant sensitizations. Molecular allergen component diagnostics (component-resolved diagnostics) should be used where appropriate to distinguish genuine sensitization from cross-reactivity and to identify major versus minor allergen components. Clinical correlation between sensitization pattern and symptom history—a patient with perennial symptoms and house dust mite sensitization represents a clearer AIT candidate than one with polysensitization and no dominant allergen-symptom relationship. Assessment of comorbid allergic rhinitis, chronic rhinosinusitis with or without nasal polyps, and atopic dermatitis is relevant because these comorbidities may independently motivate AIT and modify the risk-benefit assessment (7, 9, 42).

### Step 3: initiate and optimize biologic therapy

Select the biologic agent most appropriate for the patient's phenotype and endotype according to approved indications and current guideline recommendations. Where AIT is a longer-term treatment goal and the patient's phenotype and eligibility criteria (including total IgE within the approved dosing range) support its use, omalizumab is the preferred biologic, given the strongest evidence base for the biologic–AIT combination.

Other biologics (e.g., anti-IL-4R*α*, anti-IL-5/5R) may be more appropriate for patients whose endotyping (blood eosinophil count, FeNO, comorbidity pattern) guides selection of an alternative agent (anti-IL-4R*α*, anti-IL-5/5R, anti-TSLP), or for those with inadequate omalizumab response.

Clinicians should be clearly aware that the evidence supporting AIT combination with non-omalizumab biologics is currently limited to mechanistic reasoning and isolated case observations. For dupilumab, anti-IL-5 agents, and tezepelumab, the decision to pursue concurrent AIT must be considered experimental, and patients should be informed accordingly.

.Allow adequate time for biologic response assessment—typically at least four to six months—before reassessing AIT candidacy. Document disease stability using validated outcomes such as ACQ or ACT, exacerbation history, lung function, oral corticosteroid exposure, and the relevant type 2 biomarkers (3, 4, 43).

### Step 4: reassess AIT candidacy under biologic therapy

After a minimum of four to six months of biologic therapy with documented clinical response, reassess whether the patient meets criteria for safe AIT initiation. As a proposed, not yet validated, starting point for clinical judgement, minimum criteria for AIT consideration might include: well-controlled asthma (ACQ ≤ 1.5 or ACT ≥ 20), FEV1 ≥ 70% predicted, no severe exacerbation in the preceding three months, no requirement for maintenance oral corticosteroids, stable comorbidities, confirmed clinically relevant monosensitization or dominant sensitization amenable to AIT, and absence of contraindications to the selected immunotherapy product. The decision should be made jointly by allergy and respiratory medicine specialists with experience in both biologic therapy and allergen immunotherapy (7, 9, 10). These thresholds should be distinguished from the biomarker cutoffs proposed by Larenas-Linnemann et al. for allergic asthma *suspicion* (FeNO ≥20 ppb, blood eosinophils ≥150/µL) (12), which serve a different diagnostic purpose — identifying candidates for allergen-driven asthma workup — rather than defining post-biologic stability for AIT initiation as proposed here. The two frameworks are complementary rather than conflicting, but their numeric non-equivalence reflects the current absence of a single, prospectively validated threshold set governing the entire biologic-to-AIT care pathway. It is to mention that clinical stability under biologic therapy reduces but does not eliminate AIT-associated risk. Residual allergen-specific effector cell reactivity persists even under effective IgE suppression, and systemic reactions during SCIT build-up remain possible. Enhanced monitoring, mandatory observation periods, and immediate availability of injectable epinephrine remain non-negotiable regardless of biologic background.

### Step 5: initiate AIT with enhanced safety monitoring

In eligible patients, initiate AIT while continuing biologic therapy. For SCIT, the build-up phase should be conducted under close specialist supervision with standard anaphylaxis precautions, appropriate observation periods, and emergency equipment. SLIT may be preferred in patients with higher perceived anaphylaxis risk or limited access to injection clinic facilities. Lung function should be checked at each SCIT visit; immunotherapy should be deferred if PEF falls below 70% predicted or if symptoms of worsened asthma are present. Ongoing monitoring should include assessment of both biologic treatment response and AIT tolerability, safety, and early signs of immunological response, such as changes in allergen-specific IgG4 and basophil activation (7, 17, 18, 32, 33).

For safety, contraindications and pre-treatment requirements should be made explicit as shown in Table 2.

**Table 2:** Proposed safety checklist before initiating allergen immunotherapy in patients with severe allergic asthma receiving biologic therapy (adapted from gurgel et al. 2024 and current AIT guidelines).

Category	Specific condition/Requirement
ABSOLUTE CONTRAINDICATIONS	
Pregnancy (G)	AIT must not be initiated during pregnancy; ongoing maintenance may be continued with caution on a case-by-case basis
Uncontrolled asthma (G)	AIT is contraindicated if asthma is inadequately controlled; biologic stabilization must precede AIT initiation
Inability to use injectable epinephrine (G)	Patient or caregiver must be able to administer epinephrine auto-injector in the event of anaphylaxis
RELATIVE CONTRAINDICATIONS — individualized risk–benefit assessment	
Concomitant beta-blocker use (G)	Beta-blockers impair epinephrine response to anaphylaxis; review indication and consider switching to an alternative agent before AIT
History of anaphylaxis (G)	Prior severe systemic reaction increases risk; requires specialist assessment and enhanced monitoring protocol
Systemic immunosuppression (E)	May alter immune response to AIT and increase infection risk; evaluate risk-benefit individually
Eosinophilic oesophagitis (SLIT only) (G)	SLIT is contraindicated in patients with confirmed eosinophilic oesophagitis; SCIT may be considered if otherwise eligible
PRE-TREATMENT SAFETY REQUIREMENTS before each AIT administration (G)	
The clinician administering AIT must be trained and equipped to diagnose and manage anaphylaxis, including immediate access to injectable epinephrine and resuscitation equipment.	
Before initiating AIT, the patient must be evaluated for signs and symptoms of asthma, including lung function assessment. AIT must not be administered if the patient shows features of uncontrolled asthma or acute bronchoconstriction.	
Before each subsequent AIT dose, the patient must be re-evaluated for asthma control. Immunotherapy should be deferred if patient shows symptoms of uncontrolled asthma (e.g., PEF or FEV₁ < 70% of the patient's personal best), or significant acute illness.	

"(G) = recommendation derived from a published clinical practice guideline (7, 9, 58). (E) = based on expert consensus or extrapolation from related clinical or pharmacological evidence in the absence of a specific guideline recommendation.

### Step 6: consider biologic tapering after sustained AIT response

After completion of a guideline-concordant AIT course, typically three years, in patients who have demonstrated sustained clinical improvement and where available, favourable immunological trends (e.g, declining IgE/IgG4 ratio or titrated skin test reactivity, Blood Eosinophil Count, BEC), noting that validated, routinely accessible biomarkers of durable tolerance are currently lacking, a structured attempt at biologic dose reduction or discontinuation under close follow-up may be considered within a prospective registry or clinical trial framework. This step must be understood as a research hypothesis with no current clinical evidence base and should not be attempted outside a prospective trial framework. The mechanistic rationale rests on the premise that AIT-induced allergen-specific tolerance — mediated by regulatory T cells, IgG4 blocking antibodies, and mast cell desensitisation — may progressively reduce the effector activation that biologic therapy was originally targeting, rendering continued pathway blockade partially redundant in selected patients. This inference, while immunologically plausible, remains unproven. Clear criteria for biologic resumption must be established in advance. The long-term goal is to determine whether biologic-enabled AIT can shift the therapeutic endpoint from disease control maintained by ongoing treatment to durable allergen-specific tolerance that persists after biologic withdrawal (40, 41, 44).

## 6 Biomarkers and patient selection

Identifying which patients with severe allergic asthma are most likely to benefit from the sequential biologic–AIT strategy requires integration of clinical, functional, and immunological parameters. No single validated biomarker currently identifies the optimal candidate, and patient selection must therefore rest on a composite assessment. However, several biomarker categories are of particular relevance (3, 42, 45).

Total and allergen-specific IgE levels remain important for biologic eligibility, particularly omalizumab dosing, and for characterizing allergic sensitization. However, total IgE alone has limited predictive value for AIT response. Component-resolved diagnostics provide superior information by distinguishing sensitization to major allergen components—such as Der p 1 and Der p 2 for house dust mite, Phl p 1 and Phl p 5 for grass pollen, Fel d 1 for cat, or Alt a 1 for Alternaria—from cross-reactive minor components and carbohydrate determinants. Sensitization predominantly to major allergen components is associated with better AIT efficacy and should be confirmed before treatment initiation (7, 42).

Fractional exhaled nitric oxide (FeNO) serves as a validated biomarker of eosinophilic airway inflammation and IL-13 activity. Elevated baseline FeNO predicts response to type 2-targeted treatment and ICS, and serial FeNO monitoring under biologic treatment can assist in assessing adequacy of inflammatory control before AIT initiation. A FeNO below 25 ppb after biologic stabilization has been proposed as a potential indicator of sufficient attenuation of airway type 2 inflammation to permit safer allergen exposure. TThis threshold is based on expert extrapolation from existing FeNO guidelines and has not been prospectively validated as a safety criterion for AIT initiation in biologic-treated patients; it should not be applied as an established clinical cutoff. However, FeNO interpretation requires consideration of confounders including atopy itself, nasal polyposis, and technical measurement factors (2, 45, 46).

Blood eosinophil count (BEC) is a widely used, guideline-recommended biomarker for biologic selection in severe asthma. A persistently elevated BEC (≥ 300 cells/*μ*L) under adequate ICS therapy supports use of anti-IL-5 pathway agents and indicates dominant eosinophilic endotype. For the combined biologic–AIT strategy, monitoring BEC normalization under biologic treatment has been proposed as a pragmatic safety signal: a BEC that remains markedly elevated despite biologic therapy may indicate insufficient inflammatory control and could prompt reconsideration of AIT timing. No prospective study has validated a specific BEC threshold for this purpose; its use as a surrogate safety signal is based on pathophysiological reasoning and expert opinion only (3, 21, 27, 28).

Basophil activation testing (BAT) has emerged as a functional biomarker of allergen-specific effector cell reactivity that is independent of IgE level and skin prick test positivity. BAT measures the allergen-triggered upregulation of CD63 or CD203c on basophil surfaces as a proxy for IgE-mediated activation. Under omalizumab therapy, basophil Fc*ε*RI expression decreases substantially, and BAT results may be attenuated. The degree of BAT suppression may serve as a pharmacodynamic indicator of free IgE reduction and may help define a safety window for AIT initiation. Conversely, monitoring the re-emergence of BAT reactivity as a marker of allergen-specific re-sensitization after biologic reduction is an area of active investigation (16, 47, 48).

Serum periostin, a downstream marker of IL-13 activity, has been studied as a predictor of biologic response but has not been validated as an AIT selection biomarker. Thymic stromal lymphopoietin (TSLP) and IL-33 levels in blood and bronchoalveolar lavage may reflect epithelial alarmin activity and could in principle serve as upstream biomarkers of environmental allergen burden, but their clinical utility in the combined treatment context requires prospective evaluation.

The development of a biomarker panel that integrates allergen-specific sensitization profile, type 2 biomarker levels, functional effector cell responsiveness, and lung function stability represents an important research priority for this field (26, 45).

None of the biomarker thresholds discussed in this section have been prospectively validated in biologic-treated patients initiating AIT. All proposed cutoffs represent expert consensus or extrapolation from adjacent clinical contexts and should be interpreted as working hypotheses requiring prospective validation, not as established clinical criteria.

An important dimension not yet addressed in the biologic–AIT literature is economic burden. Both treatment components are costly, and no cost-effectiveness analyses exist for their sequential combination. Whether the anticipated clinical benefits translate into a favourable incremental cost-effectiveness ratio relative to biologic monotherapy is unknown. Future prospective trials should therefore incorporate health economic analyses, including QALY modelling and healthcare resource utilisation, as mandatory endpoints.

## 7 Infection risk, antiviral immunity, and microbiome considerations

Infection risk represents a critical safety consideration in any strategy involving two immunomodulatory treatments simultaneously. Type 2 inflammation in the airway, while pathological in the context of allergic asthma, contributes to barrier function and certain aspects of mucosal immunity. Biologic suppression of type 2 pathways may therefore have bidirectional consequences: reducing immunopathology on one hand while potentially attenuating protective type 2 responses to certain parasitic infections or specific fungal pathogens on the other (13, 14).

Antiviral immunity is particularly relevant in the context of severe asthma because viral respiratory tract infections-especially rhinovirus and respiratory syncytial virus-are among the most common triggers of asthma exacerbations. IgE-bearing plasmacytoid dendritic cells contribute to early type I interferon responses through Fc*ε*RI-mediated activation, and impairment of this mechanism under omalizumab therapy has been a theoretical concern. Observational data and *post-hoc* analyses do not demonstrate a significant increase in serious viral infections with omalizumab, and some studies suggest that omalizumab may reduce exacerbations triggered by viral infections. Dupilumab treatment may also have neutral or beneficial effects on viral infection susceptibility, given that IL-4/IL-13 signalling contributes to type 2 airway epithelial dysfunction. Tezepelumab, by blocking TSLP, may preserve epithelial antiviral competence, although no clinical evidence of increased viral infection susceptibility has emerged from NAVIGATOR trial safety data (24, 25, 49–51).

Corticosteroid-sparing effects of biologic therapy are directly relevant to infection risk. Maintenance oral corticosteroids (mOCS), used in a proportion of severe asthma patients prior to biologic initiation, are associated with a broad spectrum of immunosuppression including impaired neutrophil function, reduced macrophage activity, and increased susceptibility to bacterial, fungal, and opportunistic infections. Biologic-mediated mOCS elimination therefore represents a net infection-risk benefit in most patients. For the combined biologic–AIT strategy, confirmation of successful mOCS cessation prior to AIT initiation is an important safety prerequisite (3, 43, 52).

The airway and gut microbiome have increasingly been recognized as modulators of immune programming in asthma. Early-life microbial exposure, respiratory microbiome composition, and gut microbiome diversity all influence type 2 immune development and the establishment of allergic sensitization. Dysbiosis of the airway microbiome—characterized in several studies by reduced microbial diversity and relative enrichment of Proteobacteria—is associated with more severe asthma phenotypes, greater exacerbation susceptibility, and potentially impaired responses to standard therapy. Whether biologic therapy modifies the airway or gut microbiome composition, and whether such modifications favour or inhibit the immune recalibration sought through AIT, are largely unexplored questions. Preliminary data in allergic disease suggest that AIT may be associated with gut microbiome shifts, but the interaction between biologic-mediated microbiome changes, AIT-associated microbiome changes, and clinical outcomes in severe asthma remains underexplored (53–56).

## 8 Research agenda

Despite the mechanistic plausibility and growing clinical interest in biologic–AIT combination strategies for severe allergic asthma, the evidence base remains fragmented, largely observational, and insufficient to support definitive treatment recommendations. A structured research agenda is required to address the critical gaps. The following priorities are proposed (3, 39–41).

First, prospective randomized controlled trials are needed to compare (a) biologic therapy alone, (b) AIT alone in eligible patients, and (c) sequential biologic-then-AIT treatment in patients with severe allergic asthma who meet defined phenotyping criteria. These trials must be adequately powered for clinical endpoints including exacerbation rate, ACQ/ACT scores, corticosteroid exposure, and FEV1, as well as mechanistic endpoints including allergen-specific IgE/IgG4 ratios, Treg frequencies, and BAT reactivity. Long-term follow-up beyond active treatment is essential to assess whether the combination strategy confers durable remission (39–41, 44).

Second, dedicated mechanistic studies should determine the net effect of dupilumab on AIT-associated immune programming rather than presupposing impairment. Studies should longitudinally assess allergen-specific IgE, IgG1, IgG4, IgA, regulatory T-cell phenotypes, basophil function, and clinical outcomes in patients receiving dupilumab, omalizumab, other biologics, or no biologic background. Persistence of benefit after treatment cessation must be evaluated separately from safety and efficacy during active combination treatment (15, 22, 23, 29, 30, 48).

Third, real-world safety registries should be established to capture adverse events, serious reactions, infection episodes, and treatment outcomes in patients receiving biologic therapy concurrent with allergen immunotherapy. Such registries, ideally multinational and linked to existing severe asthma databases such as the International Severe Asthma Registry (ISAR) and the German Asthma Net (GAN), would enable pharmacovigilance and generate hypothesis-generating datasets to inform trial design. Standardized phenotyping criteria, biologic agent and dose data, AIT product and route information, and structured safety outcome definitions should be incorporated into registry data collection instruments (43, 57).

Fourth, the definition and validation of biomarker panels for patient selection and treatment monitoring should be a dedicated research priority. Current selection is necessarily clinical; future selection should integrate molecular biomarkers predictive of AIT responsiveness in the biologic-treated context, safety biomarkers for monitoring systemic reaction risk, and immunological response biomarkers for early identification of tolerogenic trajectories. Patient-reported outcomes, quality of life measures, and health economic analyses should be incorporated into all prospective studies to enable comprehensive evaluation of the biologic–AIT strategy (42, 45, 48).

Fifth, microbiome investigations should be embedded in prospective clinical studies to examine whether gut and airway microbial composition predicts AIT response in the biologic-treated context, whether biologic therapy modifies microbiome composition in ways that are relevant to immune tolerization, and whether targeted microbiome interventions could optimize the immunological environment for AIT. The interaction between TSLP blockade and mucosal microbial community structure is particularly underexplored and may be relevant to understanding why some patients with broad type 2 suppression nonetheless continue to experience allergen-triggered symptoms (53–56).

Sixth, health economic evaluation should be embedded in future prospective trials. Given the substantial combined cost of biologic therapy and allergen immunotherapy, demonstration of clinical benefit alone will be insufficient to support health system adoption without accompanying cost-effectiveness evidence.

## 9 Discussion and conclusion

The strongest interpretation of the current evidence is neither that biologic-enabled AIT should become routine nor that it should be dismissed as speculative. Rather, it should be understood as a plausible precision-medicine strategy for selected patients with severe allergic asthma. The rationale is especially strong when the allergic component is clinically dominant, when biologic therapy produces sustained control, and when the patient continues to have meaningful allergen-driven disease burden despite optimized asthma care (4, 7, 39).

The concept also reframes how disease modification is discussed in severe asthma. Biologics have made it possible to achieve levels of control that were previously unrealistic for many patients with severe type 2 disease. This new stability may permit evaluation of AIT in patients who would previously have been excluded because of safety concerns. In this model, biologics are not simply alternatives to AIT; they may act as enablers of later allergen-directed tolerance induction (3, 7, 8, 40).

Severe allergic asthma management should therefore evolve from isolated pathway suppression toward strategic immune reprogramming. Biologics have created a therapeutic landscape in which patients previously considered too unstable for allergen-directed intervention may become candidates for carefully monitored allergen immunotherapy. In selected individuals, this may open a therapeutic window for shifting the goal from short-term disease control to long-term allergen-specific tolerance. The concept of ‘control first, tolerance second' should be tested prospectively as a precision-medicine strategy for severe allergic asthma, with integrated assessment of safety, biomarkers, infection risk, mucosal immunity, microbiome ecology, and durable remission (3, 4, 7, 40).

The evidence base of this review has important limitations regarding generalisability. Most data derive from European specialist centres, predominantly adult White populations treated with omalizumab. Applicability to primary care settings, low- and middle-income countries, and ethnically diverse populations cannot be assumed. Future trials should explicitly recruit diverse populations and include implementation research components.
